# Clinicians Beware, Ewing’s Sarcoma After 60 Is Elusive and Rare!

**DOI:** 10.7759/cureus.6768

**Published:** 2020-01-24

**Authors:** Dean Davis, Ethan Berg, Eukesh Ranjit, Priyanka Bhandari, Amit Sapra

**Affiliations:** 1 Family Medicine, Southern Illinois University School of Medicine, Springfield, USA

**Keywords:** ewing's, sarcoma, adult, bone tumors, rare presentation, pain control, metastatic disease, back pain, elder

## Abstract

Ewing’s sarcoma is the second most common malignant bone tumor in children, with the worst outcomes seen in patients over the age of 20. However, the onset of the disease is much less common in people over the age of 30. This case represents the diagnostic dilemma posed by an otherwise “straight forward” case of back pain. Keeping the differential diagnosis sufficiently broad to include bone malignancies, so as not to delay diagnosis and treatment, provides the best chance at a positive outcome.

## Introduction

Ewing’s sarcoma is the second most common malignant bone tumor in children and young adults, and it accounts for 10-15% of all primary bone tumors [1-3]. Its annual incidence is 0.6/million, and it is more common in males compared to females with a 1.5:1 incidence ratio [3]. In the UK, Ewing’s sarcoma affects 13 per million people under the age of 24 annually [3]. In North America, approximately 255 cases of Ewing’s sarcoma are diagnosed per annum in people under 20 years of age. Its estimated incidence is one in 100,000 among patients aged 10 to 19 years, and it is more common in whites and Asians than in blacks per the National Cancer Institute [4]. Ewing's sarcoma and osteosarcoma have similar characteristics, with Ewing’s sarcoma primarily affecting children and adolescents, with a median age of 15 years [5,6]. Ewing's sarcomas typically develop in the pelvis, diaphysis of long bones, ribs, and scapula, and they usually metastasize to the lungs and other bones as well [4]. Primary sites of Ewing’s sarcoma include the following: lower extremity (41%), pelvis (26%), chest wall (16%), upper extremity (9%), spine (6%), hand and foot (3%), skull (2%) [7]. 

The actual incidence of Ewing’s sarcoma in the elderly is rare and not well known. However, a recent report of three cases by Xu et al. does support the potential for this diagnosis in the elderly population [8]. In 2018, Liu et al. examined clinical features and prognostic factors in Ewing’s Sarcoma patients over the age of 40 from 1973 to 2015, and were able to obtain 162 cases from the Surveillance, Epidemiology, and End Results database [9]. 

The cell origin of Ewing’s sarcoma is unknown. These tumors have been hypothesized to arise from undifferentiated, primitive neural crest or neuroectodermal cells. Recently, it has been found that the cells of origin of Ewing’s sarcoma are primitive stem cells, and the stage of stem cell arrest during differentiation determines the degree of malignancy [7]. Ewing’s sarcoma is included in a group of tumors known as small blue round cell tumors based on their microscopic features [7]. 

Pathognomonic translocations involving the EWS gene on chromosome 22 and an ETS-type gene on chromosome 11, are implicated in many but not all cases. However, the genetic etiology of Ewing’s sarcoma is beyond the scope of this article and is best suited for a formal review article.

Given the relative rarity of this entity and the value of early diagnosis providing a significant improvement in outcome for these patients, it is prudent for primary care providers to keep this diagnosis in the differential diagnosis.

## Case presentation

The patient was a 61-year-old male who presented to the primary care clinic to establish care. The patient reported non-urgent lower back pain of abrupt onset after pulling a pin on commercial motor vehicle while positioned awkwardly. The initial clinical evaluation was consistent with musculoskeletal sprain/strain. 

Past medical history included hypertension, diabetes mellitus, sleep apnea, obesity, alcohol abuse. The patient also had a remote history of gastric mucosa-associated lymphoid tissue (MALT) lymphoma stage 1E in 2004 (status post partial gastrectomy) with recurrence in 2011 for which he underwent six cycles of rituximab, cyclophosphamide, doxorubicin hydrochloride, vincristine (Oncovin), and prednisone (R-CHOP) with complete resolution of disease on positron emission tomography (PET)-CT in 2012. He started Rituxan therapy, and this was scheduled for every three months for two years. However, due to financial reasons, he received four doses of Rituxan only. 

**Figure 1:**
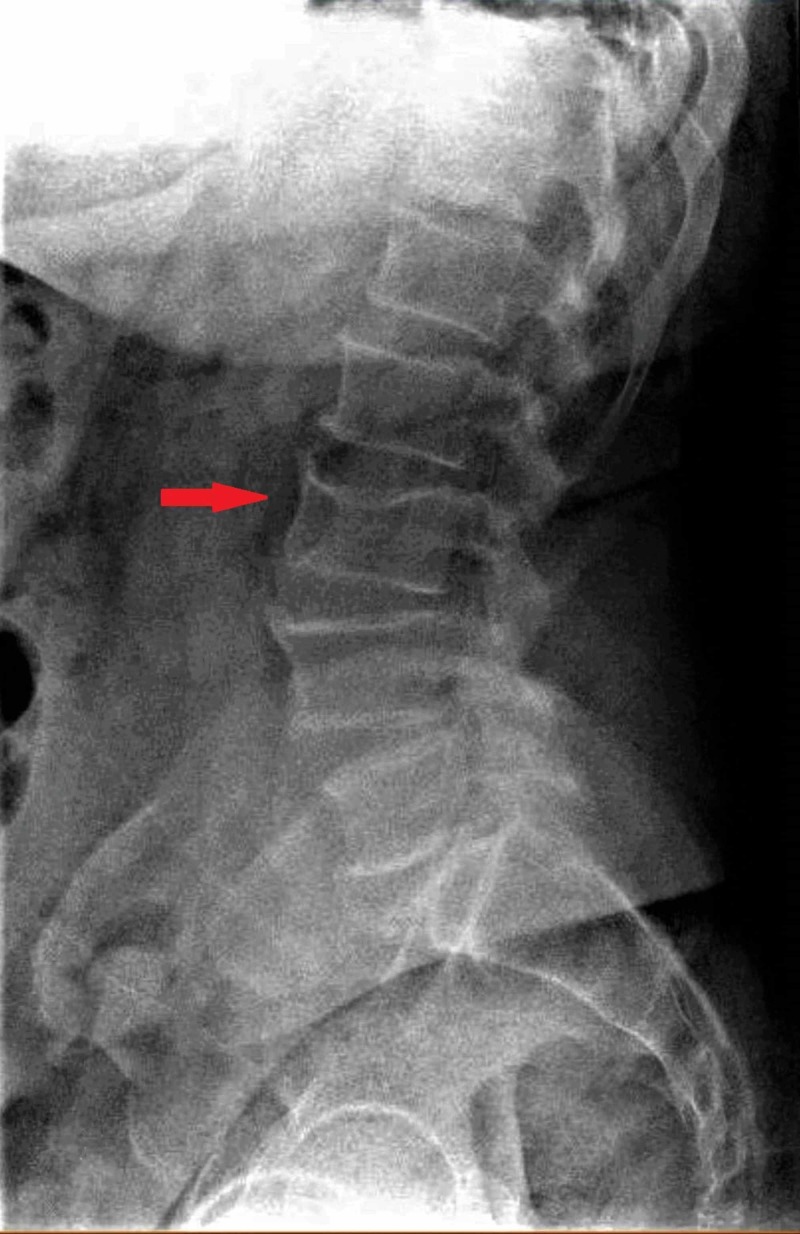
Lateral view of the lumbar spine X-ray showing L3 vertebral deformity

Unfortunately, this patient did not have relief from his back pain in the expected time course and was re-evaluated. At the time of re-evaluation, two weeks after the initial presentation to the primary care provider, he was noted to have focal tenderness to palpation overlying the lower thoracic region and no longer was experiencing a muscle spasm. Plain radiographs of the lumbar and thoracic vertebrae revealed a lytic lesion (Figure 1).

**Figure 2:**
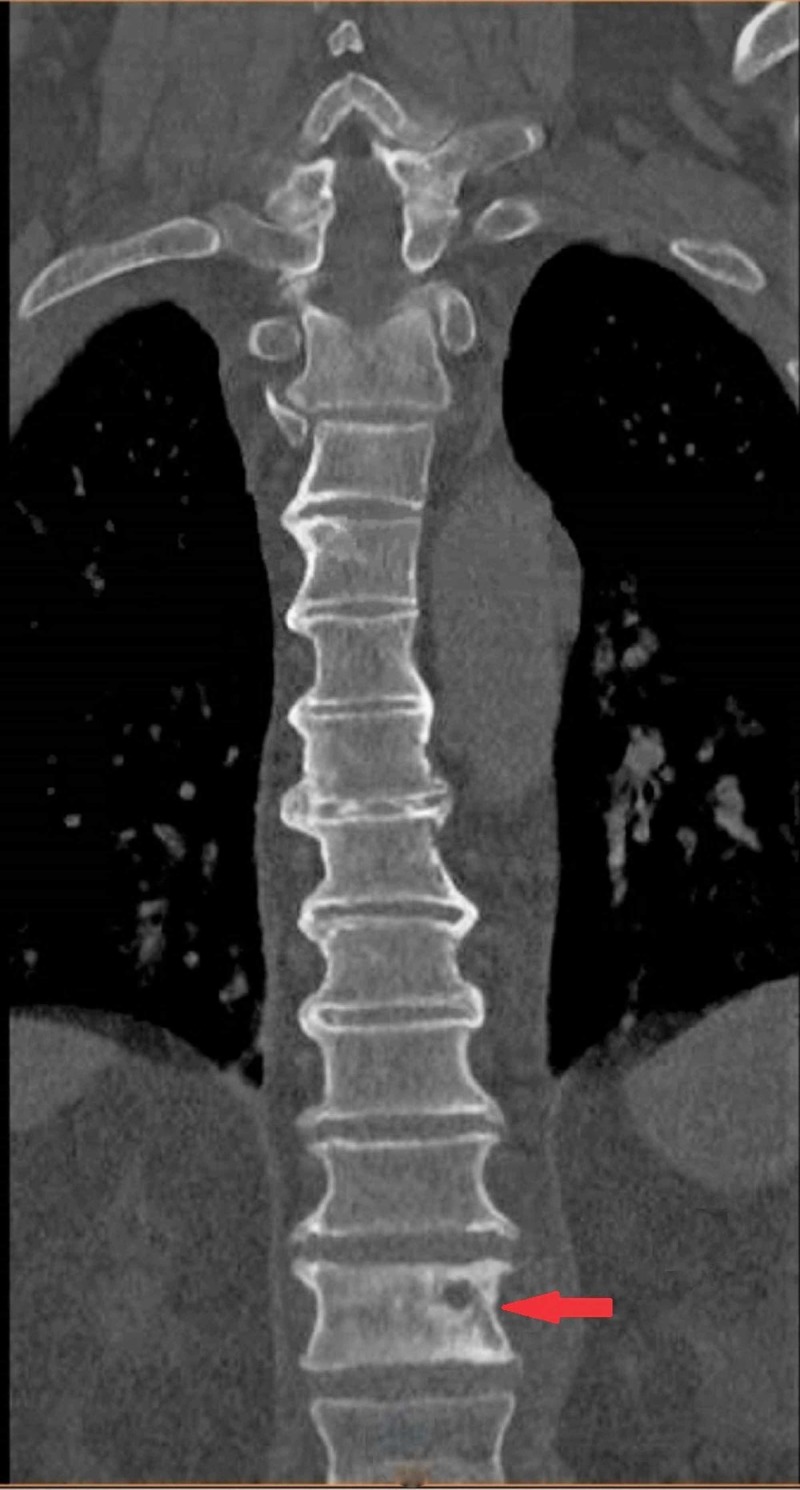
MRI lumbar spine coronal section demonstrating the involvement of the L3 vertebra

**Figure 3:**
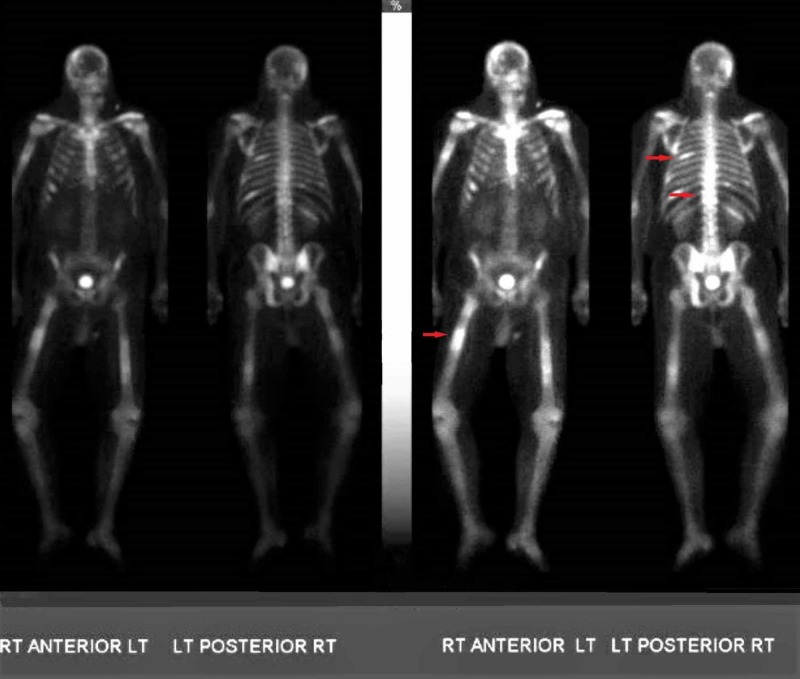
The PET-CT scan showing hypermetabolic activity involving the T12 vertebral body and paravertebral soft tissues, right femoral shaft, and left sixth rib

MRI and PET-CT scans were ordered, and referral to oncology was obtained (Figure 2). The PET-CT scan showed hypermetabolic activity involving the T12 vertebral body and paravertebral soft tissues, right femoral shaft, and left sixth rib (Figure 3).

**Figure 4:**
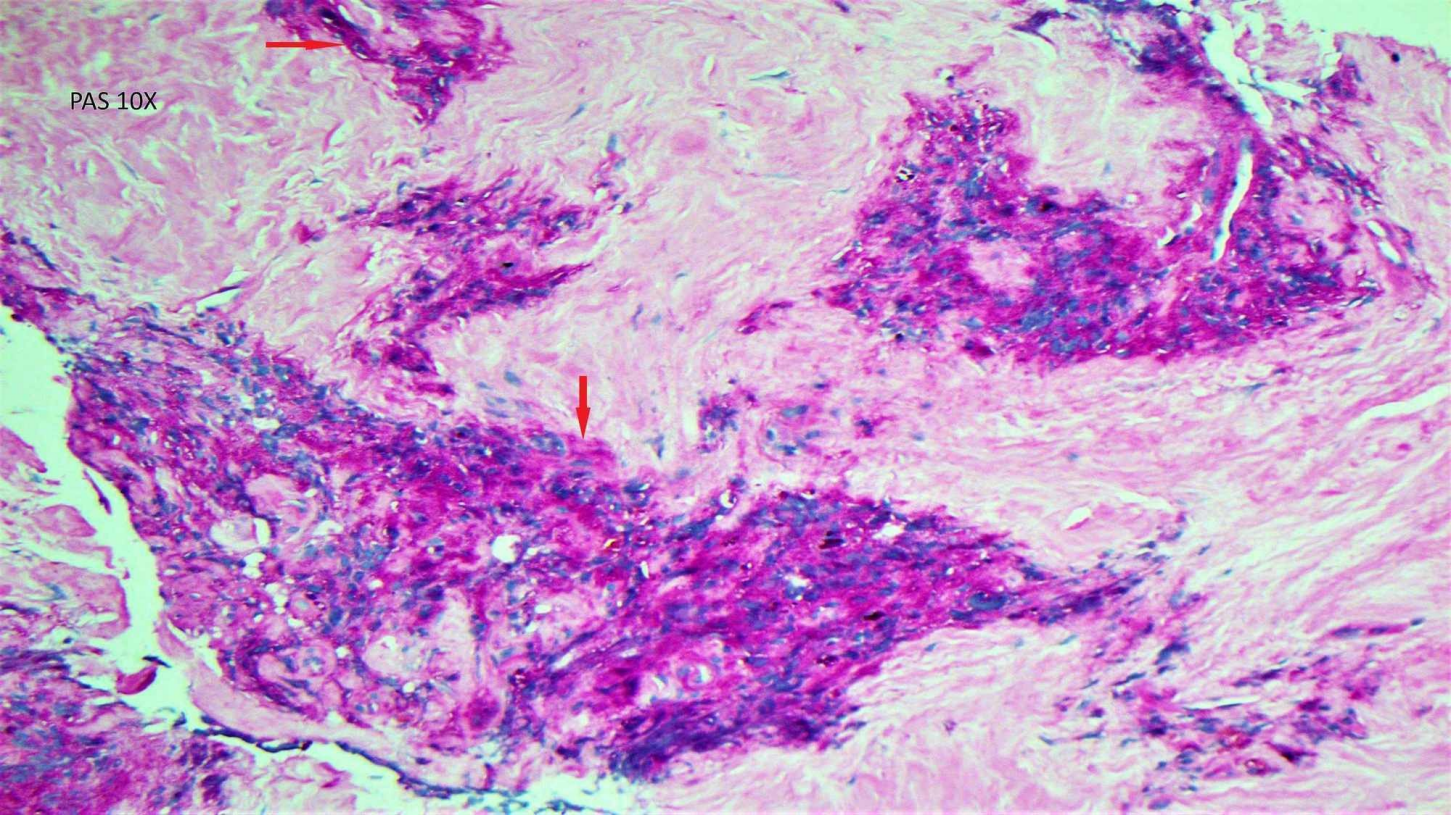
Histopathology slide showing PAS stain positive cells PAS- periodic acid-Schiff

Biopsy of the T12 lesion, performed by the oncology service, revealed blood and osseous material without evidence of malignancy. A paraspinal soft tissue mass biopsy showed neoplastic cells positive for BCL2 and CD99 (GenPath) and rare cells positive for CKAE1/AE3, and special staining for periodic acid-Schiff (PAS) stain showing cells with glycogen (Figure 4).

This sample had no distinct immunoreactivity for S100, Desmin, CD45, PAX5, CD56, SOX10, HMB45, CD20, WT1, CD34, CD3, CD10, or Synaptophysin. The molecular testing for a definitive diagnosis of Ewing’s sarcoma as the EWSR1 bracket part arrangement by fluorescence in-situ hybridization (FISH) was negative. The morphologic features favored Ewing’s sarcoma. Subsequently, he was referred to a tertiary facility for a second opinion and another biopsy of a separate osseous lesion. The diagnostic bone biopsy obtained at this facility was consistent with Ewing's sarcoma.

**Figure 5:**
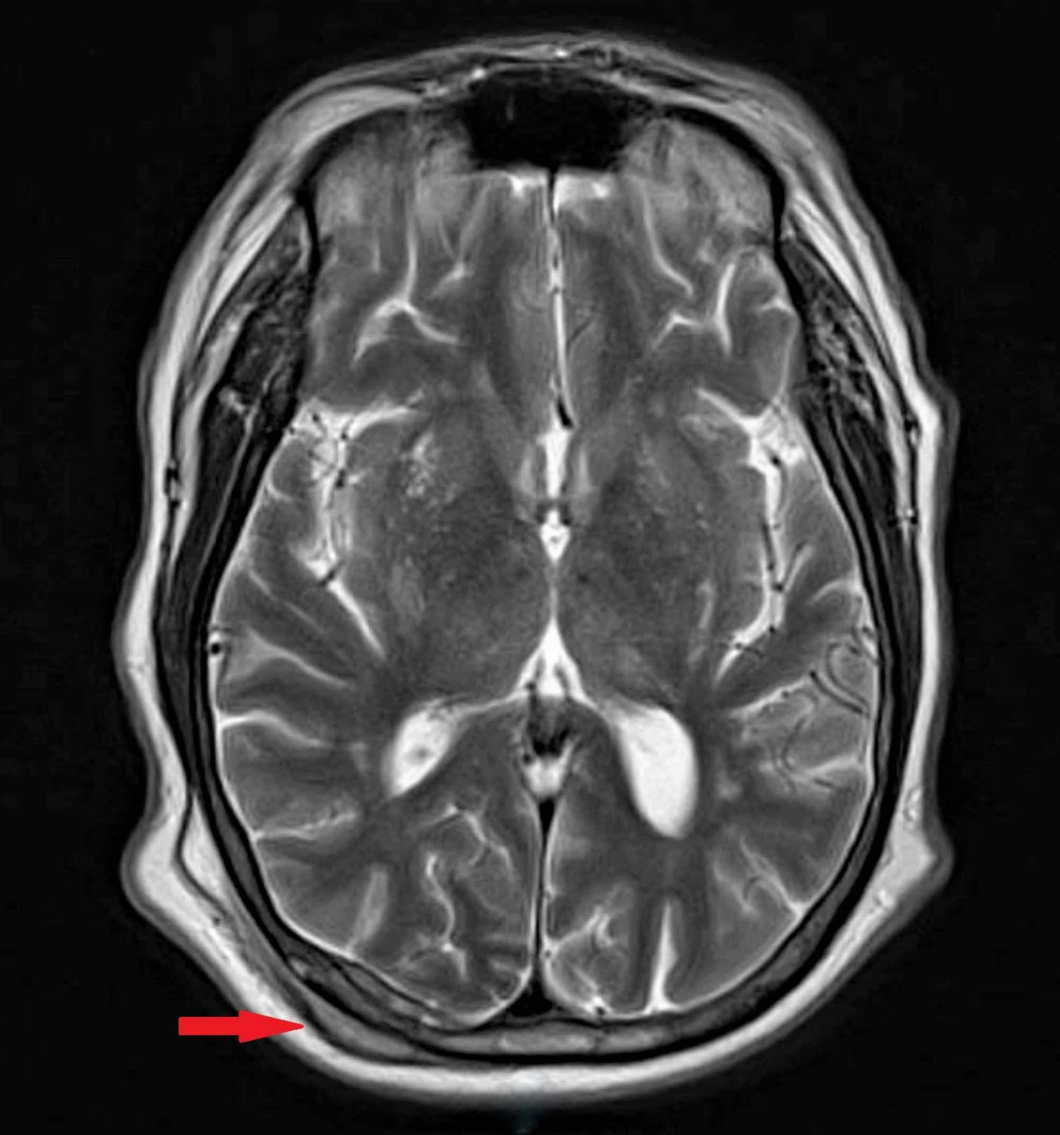
Metastasis involving calvarium along the right occipital bone

He was scheduled to see a sarcoma specialist. However, he was admitted to a local facility for pain control. He was found to have lesions involving cranium, right humerus, several ribs, right hepatic lobe involvement, multiple lesions to the pelvis and femoral neck requiring intravenous morphine (Figure 5).

The goals of care were discussed with his daughter, who at that time was yet to be named power of attorney and was consulted as the surrogate decision-maker. The decision was made to initiate chemotherapy with one cycle of vincristine, doxorubicin, and cyclophosphamide, at the patient's and family's request. The patient had marked improvement with a significant reduction of pain and analgesic requirements. He was discharged from the hospital with oral analgesics and was seen by a sarcoma specialist. At the two-week hospital follow-up visit, the patient was in his usual jovial spirit and continued to have decreased need for opioid analgesics.

He was continued on vincristine, doxorubicin, and cyclophosphamide with the addition of Zinecard (dexrazoxane) for cardio-protective effects. However, he did suffer from a 16% reduction in ejection fraction noted on the multigated acquisition (MUGA) scan after four cycles of chemotherapy. Thus, doxorubicin was discontinued and replaced by dactinomycin. After his initial response to treatment, he was noted to have disease progression and was transitioned to high dose ifosfamide and etoposide.

Over several months following the initial diagnosis, he was admitted for neutropenic fever, cholangitis, and pain control on several occasions. Eventually, his pain became increasingly difficult to control, and the patient needed to ingest a total of 450 morphine units daily for analgesia. After multiple discussions with the patient and family, he was transitioned to hospice care and succumbed to his disease process.

## Discussion

In this particular case, the EWS translocation did not meet the local lab cut off of 20% penetrance by FISH. However, repeated sampling of multiple lesions did render the diagnosis of Ewing’s sarcoma. This case demonstrates that although Ewing’s sarcoma is rare in the elder adult population, it can occur at any age, and the importance of a broad differential is paramount. Particularly in patients with lytic bone lesions, Ewing’s sarcoma should be included in the differential. Also, there is a need to differentiate Ewing’s sarcoma from other primitive neuroectodermal tumors (PNETs) for prognostication and treatment. This case elicits that by keeping a broad differential, prompt diagnosis can be made and treatment initiated with the hopes of giving the best outcome.

Current therapy for Ewing’s sarcoma combines both surgical resection up to and including limb amputation and chemotherapy. The most ominous negative prognostic factor for survival is the presence of metastatic disease, particularly non-pulmonary metastasis [5,9]. Evidence also seems to show a difference in tumor location by age with elder adult patients having more axial skeletal involvement and children and young adults having more involvement in the lower extremities [9-11]. 

The literature reveals a paucity of information regarding Ewing’s sarcoma in the elderly, likely due to the low incidence rate of this disease in this population. In this instance, keeping a sufficiently broad differential diagnosis allowed this diagnosis to be reached. This begs the question, would the incidence of Ewing’s sarcoma be higher if it was included more often in the differential diagnosis of elder individuals with lytic bone lesions? For now, we may only speculate as to the actual incidence of Ewing’s sarcoma in the elder adult population and strive for the perfection of diagnostic accuracy.

## Conclusions

Extraskeletal Ewing’s sarcoma is a rare malignant neoplasm that has been reported to involve most frequently the soft tissues of the lower extremity and the paravertebral region. The incidence of Ewing's sarcoma in the elder adult population is very low; however, the actual incidence remains unclear. This particular case reminds clinicians to maintain a broad differential diagnosis to care for adults with lytic bone lesions properly.
